# The impact of interrupted corneal collagen crosslinking (CXL) treatment

**DOI:** 10.1007/s00417-024-06505-x

**Published:** 2024-06-18

**Authors:** Marit Tholvsen, Karim Makdoumi

**Affiliations:** 1 Department of Ophthalmology, Falu Hospital, Falun, Sweden; 2 Center for Clinical Research, Dalarna, Uppsala University, Uppsala, Sweden; 3 Department of Ophthalmology, Faculty of Medicine and Health, Orebro University, Orebro, Sweden

**Keywords:** Keratoconus, Corneal collagen crosslinking, CXL, Thin cornea, Interrupted

## Abstract

**Purpose:**

To evaluate progression of keratoconus in patients where CXL treatment was interrupted due to insufficient swelling of the cornea.

**Methods:**

A retrospective review was conducted of all patients with keratoconus diagnosis who underwent CXL at the Department of Ophthalmology, Örebro University Hospital (USÖ) during the years 2010–2017. In total 377 eyes of 280 patients were screened for inclusion. In 17 eyes (15 patients), the treatment was interrupted due to insufficient swelling of the cornea. Patient journals were reviewed and keratometry examinations were analysed for long-term progression.

**Results:**

Eleven eyes (nine patients) were included in the study. Five eyes showed no signs of progression after the interrupted CXL treatment. In one eye progression continued, however, first after a period of a number of years, indicating a delayed course of clinical progression.

**Conclusion:**

This study indicates that debridement of the corneal epithelium and riboflavin administration without intense UVA radiation may slow or arrest the progression of keratoconus, likely due to photosensitisation from ambient light.

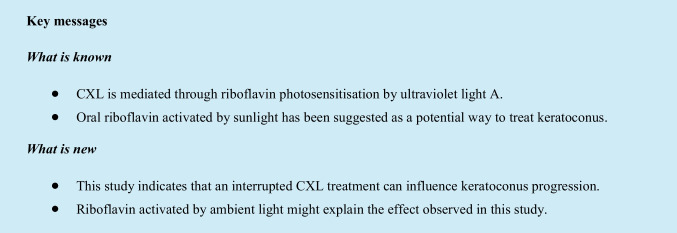

## Background

Keratoconus is a non-inflammatory progressive corneal ectasia. As a result of the reduced biomechanical rigidity the cornea is destabilised with thinning, protrusion and increased myopia often with irregular astigmatism as characteristic findings [1, 2]. Although the condition is usually bilateral, the expression is usually asymmetric. The underlying pathogenesis is complex with both environmental and genetic factors linked to the development and progression of the disease [1].

Corneal crosslinking (CXL) is used to prevent progression of keratoconus. The original treatment protocol was developed in Dresden, Germany, and the first clinical results were published in 2003 [3]. The treatment generates oxidative stress, which induces an augmented biomechanical strength of the weakened corneal stroma. Through a combination of riboflavin (vitamin B_2_) and ultraviolet A radiation new covalent bonds are formed in the stroma and the progression of keratoconus is often arrested [4, 5].

The original crosslinking treatment protocol has been thoroughly studied and evaluated and the treatment is safe with few reported complications [6–9]. Several variations of the original method have been proposed and studied clinically in order to decrease discomfort, treatment time, and complications associated with the treatment [10–14]. To ensure the protection of the corneal endothelium from excessive UV exposure, the corneal thickness has to exceed 400 µm. If this is not the case, hypotonic riboflavin solutions can be used to swell the tissue. In a few cases, the UVA irradiation cannot take place due to insufficient swelling of the cornea and the treatment is interrupted after riboflavin application.

In order to manage CXL in keratoconic eyes with thin corneas several other concepts have been proposed and evaluated clinically, such as treatment using transepithelial CXL, leaving the epithelium over the corneal apex or reducing the stromal exposure by UVA by applying a contact lens, a lenticule from SMILE procedure [15] as well as customised irradiation based on corneal thickness [16, 17]. Oral riboflavin in combination with direct sunlight exposure has also been suggested as an alternative approach to halt the progression of keratoconus [18].

The aim of the study was to examine if progression of keratoconus is influenced after interrupted CXL treatment due to insufficient swelling of the cornea. Since these eyes have received the photosensitiser (riboflavin) after removal of the corneal epithelium, it is possible sthat surrounding background light can mediate enough oxidative stress to influence the progression of keratoconus.

## Material and methods

The study adhered to the tenets of the declaration of Helsinki and was approved by the regional review board. Written informed consent was obtained from all subjects included in the study.

The medical charts were reviewed for all patients diagnosed with keratoconus (ICD diagnosis code H18.6) who underwent CXL (CGD99) during the years 2010–2017 at the Department of Ophthalmology at Örebro University Hospital, Sweden. A total of 377 from 280 patients were screened for inclusion. Treatment was discontinued in 17 eyes, of 15 patients, due to insufficient corneal thickness. Patients with post-LASIK or pellucid marginal degeneration (PMD) were excluded.

Corneal tomography examinations (Pentacam, Oculus Optikgeräte GmbH, Wetzlar, Germany) were stored in the system database, and parameters from the preoperative examination were compared with data from the last visit or when clinical progression was assessed. Excluded were patients who did not cooperate at examination with the Pentacam and thus no comparative data was available. Measurements from cases, which were initially examined using corneal topography (Orbscan II, Bausch & Lomb, Rochester, New York, USA) and later followed by corneal tomography, due to equipment exchange, were acquired but this data was not included in the final interpretation.

Clinical diagnosis considered Kmax, K_1_, K_2_, astigmatism, and visual acuity, as well as slit-lamp examination in combination with the clinical symptoms described by the patient. In all cases, clinical progression was supported by changes in refraction from optician/optometrist, such as increased myopia and astigmatism, fulfilling the indications for CXL [19].

There is a lack of consensus in which indices to take into consideration in determining progression of keratoconus [19]. The primary outcome measurement in this study was Kmax (maximum keratometry front). The progression was considered arrested when the Kmax increase was ≤ 1 dioptre (D) for a duration of 12 months or longer. However, clinical assessment of the condition and secondary outcome measures parameters were also included such as K_1_ and K_2_, corneal astigmatism and thinnest pachymetry as well as uncorrected visual acuity (UCVA) and best corrected visual acuity (BCVA). Keratoconus stage was classified according to the modified Krumeich scale [20]. Given the retrospective data collection, not all parameters were available in every case studied.

In all eyes included, both hypotonic riboflavin solution and sterile water were administered during the attempted CXL procedure to reach sufficient corneal thickness. Irradiation with UVA was not conducted since adequate corneal thickness could not be achieved in order to avoid corneal endothelial cell loss. Postoperatively, antibiotics (levofloxacin 5 mg/ml) and anaesthetic eye drops (alcaine 5 mg/ml) were prescribed. No bandage contact lens was applied during epithelial healing.

## Results

Fifteen patients met the inclusion criteria; however, four of these were not included due to lack of data from before the attempted treatment, poor cooperation at the preoperative visit or missing follow-up visit or Pentacam examination. Furthermore, two patients declined inclusion in the study. Hence, 9 patients with a total number of 11 eyes were included in analysis, six men and three women. Median age was 22 years (range 17–29 years; mean 22 ± 4 months) at the time of attempted CXL. See Table 1 for specific information on each patient.

**Table 1:** Preoperative data D = dioptres

Included patients	9
Age (years)	
Mean	22 ± 4
Range	17–29
Median	22
Gender mean	f:m 19:24
Gender	
Female	3
Male	6
Atopic/allergy	4
Eye rubbing	1
Developmental delay	1
Other disease	0
**Baseline keratometry values**	
Pachymetry (µm)*	
Mean	395 ± 55
Median	394 (307–498)
Kmax (D)†	
Mean	61 ± 5
Median	61

11 eyes, values < 6 months prior to CXL

^†^6 eyes with available values from Pentacam

In the subgroup of eyes where keratoconus progression was identified, it was diagnosed at a median time 18 months (range 3–92 months; mean 28 ± 33 months) after the attempted treatment.

Three eyes showed progression within one year after the attempted CXL treatment, without any signs indicating reduced keratoconus progression two of which were operated with deep anterior lamellar keratoplasty (DALK) procedure. In two additional eyes, progression was established after 26 and 28 months respectively, indicating no apparent influence on progression. Both of these eyes, belonging to the same patient, underwent another CXL treatment in which the illumination of the cornea was successful.

One eye appeared stable after the attempted treatment during repeated examinations and did not show signs of progression for 34 months after the initial treatment attempt. However, clinical progression was subsequently confirmed after 7.5 years. Another eye developed retinal detachment and underwent a pars plana vitrectomy (PPV), followed by cataract surgery. Later on, it developed myopic choroidal neovascularisation. In this eye no sign of keratoconus progression could be detected, although refractive but visual acuity was low due to other ocular conditions.

At last follow-up visit, 5 out of 11 eyes, did not show signs of clinical progression. Among the eyes that were considered stable, three Kmax values were slightly decreased compared to the value obtained at diagnosis, over the follow-up period, which for these eyes ranged between 13 and 54 months (median 25 months). In one of these eyes no Kmax value was registered at the preoperative evaluation but two years postoperatively no clinical sign of progression was observed. Corneal astigmatism was unchanged in two eyes and reduced in three. The pachymetry values were in all cases lower than at baseline with a median reduction of 17 µm (range 3–47 µm), but did not show continued thinning during the follow-up period. The progression of keratoconus was, hence, interpreted as arrested. However, three of these patients showed progression of keratoconus in the fellow eye, in which CXL was successfully performed. Data with parameters on each case are found in Table 2.

**Table 2:** Eleven included eyes. All keratometric values measured with Pentacam unless Orb noted

Eye	K1	K2	Kmax	Ast	Pach	Stage	UCVA	BCVA	Time	Comment
1.*	59.4	62.2	66.5	2.7	360	3	0.01	0.03	Before	Male 27 years. Fellow eye CXL
	60.7	61.2	64.9	0.5	313	−	0.03	0.03	4.5 yrs	PPV due to RRD. PEI. Myopic CNVM
2.*	44.2	46.5	51.9	2.3	498	1	0.3	0.8	Before	Female 22 years. Fellow eye CXL
	44.7	47.2	51.2	2.5	481	-	0.3	0.8	3.5 yrs	
3	47.8	52.4	61.1	4.6	414	2	0.5	0.5	Before	Male 23 years. Fellow eye #4
	51.5	54.0	61.5	2.5	393	−	−	0.4	28 mos	New completed CXL performed
4	44.5	49.3	58.5	4.8	421	2	0.5	0.6	Before	Male 23 years. Fellow eye #3
	50.2	52.4	61.5	2.2	399	−	−	0.6	26 mos	New CXL performed
5	55.1(kmin)		58.9 Orb	3.9 (simK)	307	3	0.15	−	Before	Male 17 years. Fellow eye #6
	62.3	64.0	77.9	1.5	337	−	0.04	−	7 mos	DALK performed
6	50.8(kmin)		58.6 Orb	7.9 (simK)	339	3	0.4	−	Before	Male 17 years. Fellow eye #5
	52.4	58.2	65.2	5.8	386	−	0.1	0.2	3 mos	DALK performed
7.*	50.2	54.9	x	4.7	364	3	0.3	−	Before	Male 19 years. Fellow eye severe keratoconus
	51.6	55.3	67.8	3.8	361	−	0.1	0.5	25 mos	
8	51.2(kmin)		58.7 Orb	7.4 (simK)	444	2	0.2	-	Before	Male 26 years. CL before. Fellow eye CXL
	54.7	61.4	69.7	6.7	407	−	0.04	0.4	7.5 yrs	No progression at 34 mos. postop
9.*	48.4	55.7	65.7	7.3	442	2	0.2	0.3	Before	Female 18 years. Fellow eye CXL
	54.0	57.2	65.0	3.2	400	−	0.03	0.3	16 mos	
10	47.7(kmin)		51.6 Orb	3.9 (SimK)	361	3	0.1	0.2	Before	Female 18 years. Fellow eye lamellar keratoplasty
	50.3	54.8	63.8	4.5	403	−	0.02	0.2	9 mos	
11.*	48.5	53.1	61.3	4.6	394	3	0.2	0.2	Before	Male 29 years
	48.5	53.2	61.3	4.8	383	−	0.1	0.25	13 mos	

*Orb* Orbcan II, *Ast* corneal astigmatism, *Pach* thinnest pachymetry, *Stage* preoperative stage according to modified Krumeich classification [20], *UCVA* uncorrected visual acuity, *BCVA* best corrected visual acuity, *Time* examination in relation to interrupted CXL. * Eyes where the progression is arrested. *PPV* pars plana vitrectomy, *RRD* rhegmatogenous retinal detachment, *PEI* phacoemulsification with intraocular lens implantation, *CNVM* choroidal neovascular membrane, *CL* contact lens

No complications occurred after the attempted CXL treatment.

## Discussion

Among the eyes scheduled for conventional CXL treatment using the Dresden protocol five out of eleven were considered stable after the treatment although no illumination was conducted. The aim of the study was to evaluate potential effect of the interrupted CXL riboflavin without direct UVA exposure. Furthermore, one additional eye was seemingly stable over a number of years before clinical progression, why the course of the disease may have been influenced by the attempted treatment but insufficient to induce a complete arrest of keratoconus progression. Given the retrospective nature of the study some data collected is not completely reliable, as is sometimes the case in advanced keratoconus, resulting in somewhat suboptimal keratometric examinations, missing data, and variable follow-up duration. Furthermore, it is very difficult to compare data originating from different devices.

Our interpretation from the clinical assessment and this retrospective review of data, is still that around half of the eyes were positively affected by only the administration of riboflavin on the de-epithelialised corneal stroma. The arrested, or in some cases delayed, progression is probably due to oxidative stress by riboflavin photosensitisation from ambient light. Although the optimum wavelength for photosensitisation is around 370 nm, riboflavin excitation can also be mediated by visible light, particularly in the blue spectrum of visible light. Since normal daylight contains both UVA and blue light it is possible that even though no ultraviolet irradiation was done adequate strengthening effect was mediated to halt the keratoconus progression in some eyes. Several different treatment modalities have been developed for the treatment of keratoconus, PMD and Post-LASIK ectasia and even though depth of stromal demarcation line, indicating treatment efficacy after CXL differ between these multiple of the suggested variations of the original Dresden protocol seems satisfactorily efficacious to arrest the disease. Given the retrospective design of this study, corneal OCT measurements could not be conducted to assess a possible demarcation line after the interrupted CXL, which apparently would have strengthened the hypothesis raised after these observations.

Although the effect of riboflavin administration on the cornea without UVA illumination is still unclear, a number of prospective randomized, sham-controlled clinical studies have investigated the efficacy of CXL. In most of them, the control group received riboflavin without epithelium debridement and no UVA radiation and the control eye was crossed over to treatment group at three or six months [9]. In a study by Sharma et al., the control group received a sham treatment with epithelium debridement followed by riboflavin drops without UVA radiation [8]. Follow-up at six months after treatment showed significant improvement in several indices in the intervention group and no change in the control group. After six months, the control group was treated with CXL, hence no long-term follow-up for these eyes.

Since the CXL treatment is normally performed successfully, even in thin corneas, the number of included eyes was low. The small number of included patients and the lack of controls are obvious weaknesses of the study. Furthermore, it is noteworthy that a number of eyes included in this study had a preoperative corneal thickness that, using the appropriate technique, should likely have been sufficient for completing the CXL treatment. Two eyes even underwent a successful treatment later. However, the study protocol evaluated all patients with keratoconus, who gave their consent, in which the CXL treatment was interrupted due to insufficient corneal thickness during the defined period. Since all subjects fulfilled these criteria, they were included in the study. During the study period, treatment failed in 17 out of 377 eyes with keratoconus due to excessively thin corneas, resulting in a failure rate of 4.5%. We consider this number relatively high; however, it is noteworthy that at the time treatment a number of innovative approaches had not yet been impemented or thoroughly evaluated.

Several alternatives to conventional CXL have been proposed to avoid endothelial cell damage in eyes with thin corneas, in addition to swelling the cornea using hypo-osmolar riboflavin. Examples include conducting transepithelial crosslinking treatment, leaving the epithelium on the apex of the cone, applying a riboflavin-soaked contact lens or using a SMILE lenticule [15, 21]. All of these methods allow exposure using a UVA lamp and ensure that proper exposure is done. Other alternatives to enable treatment of thin corneas include customised irradiation settings, as proposed through a theoretical treatment algorithm [16, 17] or using oral riboflavin to allow photosensitisation of riboflavin from surrounding daylight [18]. However, long-term data and larger patient studies are still needed to adequately assess the optimum protocol in thin corneas.

The findings reported here indicate that even an interrupted CXL treatment without exposure of UVA, likely mediated by ambient light excitation of riboflavin may, may in some eyes be sufficient to halt the keratoconus progression. Although we do not suggest the application of riboflavin on the de-epithelialised corneal stroma as a therapeutic alternative approach to CXL in thin corneas, the specific dose of oxidative stress required to arrest disease is probably individual. In cases of an unsuccessful or incomplete crosslinking treatment the patient should likely be followed for signs of further progression before other alternative options are considered. As only a small proportion of eyes with keratoconus will not be possible to treat with CXL given the more modern approaches in thin corneas, additional studies with long-term data are needed to validate these findings.
